# Cost-Effectiveness of Nivolumab Plus Ipilimumab With and Without Chemotherapy for Advanced Non-Small Cell Lung Cancer

**DOI:** 10.3389/fonc.2021.760686

**Published:** 2021-12-09

**Authors:** Szu-Chun Yang, Natalia Kunst, Cary P. Gross, Jung-Der Wang, Wu-Chou Su, Shi-Yi Wang

**Affiliations:** ^1^ Department of Chronic Disease Epidemiology, Yale University School of Public Health, New Haven, CT, United States; ^2^ Department of Internal Medicine, National Cheng Kung University Hospital, College of Medicine, National Cheng Kung University, Tainan, Taiwan; ^3^ Harvard Pilgrim Health Care Institute and Harvard Medical School, Boston, MA, United States; ^4^ Cancer Outcomes, Public Policy, and Effectiveness Research (COPPER) Center, Yale Cancer Center and Yale University School of Medicine, New Haven, CT, United States; ^5^ Public Health Modeling Unit, Yale University School of Public Health, New Haven, CT, United States; ^6^ Department of Oncology, National Cheng Kung University Hospital, College of Medicine, National Cheng Kung University, Tainan, Taiwan

**Keywords:** cost-effectiveness, immunotherapy, non-small cell lung cancer, nivolumab, ipilimumab

## Abstract

**Background:**

First-line treatment with nivolumab plus ipilimumab (N+I) or nivolumab plus ipilimumab with two cycles of chemotherapy (N+I+chemotherapy) improve overall survival and progression-free survival for patients with metastatic non-small cell lung cancer (NSCLC), yet researchers have not concomitantly compared the cost-effectiveness of N+I and N+I+chemotherapy with chemotherapy alone.

**Materials and methods:**

Using outcomes data from the CheckMate 227 and CheckMate 9LA phase 3 randomized trials, we developed a Markov model with lifetime horizon to compare the costs and effectiveness of N+I and N+I+chemotherapy versus chemotherapy from the U.S. health care sector perspective. Subgroup analysis by programmed death-ligand 1 (PD-L1) expression levels (≥1% and <1%) and probabilistic analysis were performed.

**Results:**

The incremental cost-effectiveness ratio (ICER) of N+I versus chemotherapy was $239,072 per QALY, and $838,198 per QALY for N+I+chemotherapy versus N+I. The ICER of N+I versus chemotherapy was $246,584 per QALY for patients with PD-L1 ≥ 1% and $185,620 per QALY for those with PD-L1 < 1%. In probabilistic analysis, N+I had a 2.6% probability of being cost-effective at a willingness-to-pay threshold of $150,000 per QALY. The probability was 0.4% for patients with PD-L1 ≥ 1% and 10.6% for patients with PD-L1 < 1%.

**Conclusion:**

First-line N+I or N+I+chemotherapy for metastatic NSCLC was not cost-effective regardless of PD-L1 expression levels from the U.S. health care sector perspective.

## Introduction

Platinum-doublet chemotherapy was historically the standard first-line treatment for patients with metastatic non-small cell lung cancer (NSCLC) whose tumors lack of epidermal growth factor receptor mutations or anaplastic lymphoma kinase translocations. Research has shown that immune checkpoint inhibitor (ICI) monotherapy for patients with tumor programmed death-ligand 1 (PD-L1) expression ≥ 50% (1), and a single-agent ICI in addition to chemotherapy (2–4) or bevacizumab plus chemotherapy (5) for patients regardless of PD-L1 levels improved overall survival and progression-free survival. However, prices tagged onto these treatments result in financial pressure on health care system.

Nivolumab and ipilimumab are monoclonal antibodies that bind to T-cell’s programmed death-1 and cytotoxic T-lymphocyte antigen 4, respectively, to restore T-cell activity against tumor cells. In 2019, the CheckMate 227 trial showed that first-line treatment with nivolumab plus ipilimumab (N+I) resulted in longer durations of overall survival and progression-free survival than chemotherapy for patients with metastatic NSCLC (6). Specifically, the CheckMate 227 results indicated that N+I were associated with improved survival in pre-specified subgroups including PD-L1 ≥ 1% and PD-L1 < 1%. In 2021, the CheckMate 9LA trial with randomization stratified by PD-L1 ≥ 1% and < 1% revealed that nivolumab plus ipilimumab with two cycles of chemotherapy (N+I+chemotherapy) provided significant improvements in overall survival and progression-free survival versus chemotherapy (7). U.S. Food and Drug Administration (FDA) approved N+I for patients with PD-L1 ≥ 1% (8), and the National Comprehensive Cancer Network panel extended their use for patients with PD-L1 < 1% (9). N+I+chemotherapy was also approved by the FDA for patients regardless of PD-L1 levels later (10).

Although several investigations have shown a single-agent ICI with or without chemotherapy to be cost-effective (11–15), double-agent ICI combinations incur more costs than single-ICI regimens. To date, three studies have estimated the cost-effectiveness of N+I versus chemotherapy and displayed different results (16–18). Another recently published study found that the incremental cost-effectiveness ratio (ICER) of N+I+chemotherapy versus chemotherapy alone was $202,275 per quality-adjusted life year (QALY) (19). However, researchers have not concomitantly compared the cost-effectiveness of chemotherapy, N+I and N+I+chemotherapy. We hypothesized that, compared with traditional platinum-doublet chemotherapy, either N+I or N+I+chemotherapy was not cost-effective regardless of tumor PD-L1 expression levels. Using a simulation model informed with the outcomes data from the CheckMate 227 and CheckMate 9LA trials, we concomitantly compare the costs and effectiveness of chemotherapy, N+I, and N+I+chemotherapy to verify our hypothesis.

## Materials and Methods

### Simulation Model

We developed a Markov model and simulated 10,000 stage IV or recurrent NSCLC patients that met the eligibility criteria for CheckMate 227 trial in base-case analysis. We first compared N+I and chemotherapy for all patients, patients with PD-L1 ≥ 1%, and patients with PD-L1 < 1%. Based on the chemotherapy group of CheckMate 227 trial, we standardized the characteristics of patients receiving N+I+chemotherapy in the CheckMate 9LA trial to compare three first-line treatment strategies: (1) platinum-doublet chemotherapy, (2) nivolumab plus ipilimumab, and (3) nivolumab plus ipilimumab with two cycles of chemotherapy. We estimated the ICERs in terms of incremental costs divided by incremental QALYs. The analysis was conducted from the U.S. health care sector perspective and a willingness-to-pay threshold of $150,000 per QALY was selected (20). **Supplementary Figure 1** shows the model structure. We assumed all simulated patients entered the model in a progression-free state and had to transit to progressive disease before death. A model cycle length of 6 weeks was chosen because ipilimumab was administered every 6 weeks and platinum-doublet chemotherapy was given every 3 weeks. We applied a lifetime horizon, half-cycle correction and an annual discount rate of 3% (21) for costs and QALYs. We used Amua software (version 0.3.0) to perform the analysis.

Three first-line treatments were administered up to disease progression. Platinum-doublet chemotherapy was allowed to be used for a maximum of 12 weeks, whereas nivolumab plus ipilimumab could be continued for a maximum of 2 years according to the trial design. Selection of first-line chemotherapy was consistent with trials. Pemetrexed plus carboplatin was used for patients with non-squamous NSCLC in both trials. For patients with squamous NSCLC, gemcitabine plus carboplatin and paclitaxel plus carboplatin were used in the CheckMate 227 and 9LA trials, respectively. We modeled subsequent treatments over time according to the trial data (**Supplementary Table 1**) (6, 7). For patients that progressed in the N+I+chemotherapy and N+I groups, gemcitabine plus carboplatin and pemetrexed plus carboplatin were used as the second-line chemotherapy for squamous and non-squamous NSCLC, respectively. Docetaxel was selected as the second-line chemotherapy regardless of tumor histology for patients progressed in the platinum-doublet chemotherapy group. If patients received immunotherapy as the second-line therapy, nivolumab was selected for three groups because it was most popularly used in the trials. Similarly, we selected erlotinib to be the second-line targeted therapy for three groups.

### Survival Estimates

We used a web-based software (WebPlotDigitizer; https://automeris.io/WebPlotDigitizer/) to extract the data points of progression-free survival and overall survival curves from the CheckMate 227 trial. Thus, the probability of progression-free state to progressive disease at each model cycle was directly derived from the trial results, and was time-dependent. Because we assumed that patients would not die without disease progression, we calibrated the probability of progressive disease to death at each model cycle to fit the overall survival curve. We compared the modeled overall survival with the trial results. Because the follow-up period of this trial was less than 4 years, we fitted the progression-free and overall survival curves with Weibull survival functions and extrapolated them to lifetime. Hoyle and Henley’s method using Excel spreadsheet and R software package (22) was implemented to derive the Weibull-extrapolated progression-free and overall survival for time-dependent transitional probabilities beyond the end of follow-up period. Based on the lifetime progression-free and overall survival of CheckMate 227 chemotherapy group, we simulated the survival of patients receiving N+I+chemotherapy by using the hazard ratios N+I+chemotherapy versus chemotherapy in CheckMate 9LA trial.

### Medical Costs Estimates

Medical costs included administration cost, drug costs, costs for management of adverse events, and cost for supportive care. All these costs were derived from relevant U.S. sources and based on the payments by the Centers for Medicare & Medicaid Services (11, 23–28). Using medical care consumer price indices (29), we inflated all costs to 2020 USD. A mean body weight of 70 kg, a mean body surface area of 1.84 m^2^, and a mean glomerular filtration rate of 73 mL/min (i.e., a 65-year-old male with mean serum creatinine of 1 mg/dL) were used to estimate the drug dosages. Details regarding the timing of infusion and the cost of each drug are shown on **Supplementary Table 2**. Adverse events considered in the model were those rated as grade 3 or 4 and reported in any grade ≥ 15% of patients in the CheckMate 227 trial. We adjusted the incidence of adverse events and second-line therapy of patients receiving N+I+chemotherapy based on the results of CheckMate 227 chemotherapy group.

### Health Utility Estimates

Utility estimates were derived from prior literature (30). We assumed the utility value of 0.79 for patients in the progression-free state who received first-line chemotherapy. Because prior research suggested that patients who received nivolumab plus ipilimumab had better utility than those who received chemotherapy, and the utility difference was 0.09 (31), we assumed that the utility value for patients receiving first-line nivolumab plus ipilimumab with and without chemotherapy in the progression-free state was 0.88. Patients in the progressive disease of three groups shared the same utility value of 0.72 for second-line therapy (30).

### Sensitivity Analyses

We performed one-way deterministic sensitivity analyses with the parameters varied within clinically plausible ranges on our baseline estimates. To explore the effect of model parameter uncertainty on the outcomes, we conducted probabilistic analyses using Monte Carlo simulation with 500 iterations. Ranges and distributions for different input parameters were detailed in **Table 1** and **Supplementary Table 3**. In contrast to our base-case analyses using the chemotherapy group in CheckMate 227 trial as the reference, we also conducted sensitivity analyses based on the survival of chemotherapy group in CheckMate 9LA trial.

**Table 1:** Input parameters for all patients.

Parameter	Baseline value	Range		Distribution	References for baseline value
		Minimum	Maximum		
Transitional probabilities	Time-dependent				Estimated from the trial and extrapolated survival curves (6)
Hazard ratios of N+I+chemotherapy versus chemotherapy^a^					
PFS	0.68				(7)
OS	0.66				(7)
Squamous in tumor histology^a^	28.0%			Dirichlet (163,419)	(6)
Grade 3/4 AEs incidence, N+I+chemotherapy^a^					
Diarrhea	4.8%	3.8%	5.7%	Beta (17,341)	(6, 7)
Rash	1.7%	1.3%	2.0%	Beta (6,352)	(6, 7)
Fatigue	5.5%	4.4%	6.6%	Beta (20,338)	(6, 7)
Decreased appetite	1.2%	0.9%	1.4%	Beta (4,354)	(6, 7)
Nausea	3.4%	2.7%	4.1%	Beta (12,346)	(6, 7)
Anemia	4.8%	3.8%	5.7%	Beta (17,341)	(6, 7)
Neutropenia	7.0%	5.6%	8.3%	Beta (25,333)	(6, 7)
Grade 3/4 AEs incidence, N+I^a^					
Diarrhea	1.7%	1.4%	2.0%	Beta (10,566)	(6)
Rash	1.6%	1.3%	1.9%	Beta (9,567)	(6)
Fatigue	1.7%	1.4%	2.0%	Beta (10,566)	(6)
Decreased appetite	0.7%	0.6%	0.8%	Beta (4,572)	(6)
Nausea	0.5%	0.4%	0.6%	Beta (3,573)	(6)
Anemia	1.4%	1.1%	1.7%	Beta (8,568)	(6)
Grade 3/4 AEs incidence, chemotherapy^a^					
Diarrhea	0.7%	0.6%	0.8%	Beta (4,566)	(6)
Fatigue	1.4%	1.1%	1.7%	Beta (8,562)	(6)
Decreased appetite	1.2%	1.0%	1.4%	Beta (7,563)	(6)
Nausea	2.1%	1.7%	2.5%	Beta (12,558)	(6)
Anemia	11.6%	9.3%	13.9%	Beta (66,504)	(6)
Neutropenia	9.5%	7.6%	11.4%	Beta (54,516)	(6)
Second-line therapy proportion, N+I+chemotherapy^a^					
Chemotherapy	38.7%	31.0%	46.4%	Beta (140,221)	(6, 7)
Immunotherapy	7.1%	5.7%	8.5%	Beta (26,335)	(6, 7)
Targeted therapy	6.5%	5.2%	7.8%	Beta (23,338)	(6, 7)
Second-line therapy proportion, N+I^a^					
Chemotherapy	35.0%	28.0%	42.0%	Beta (204,379)	(6)
Immunotherapy	5.5%	4.4%	6.6%	Beta (32,551)	(6)
Targeted therapy	5.7%	4.6%	6.8%	Beta (33,550)	(6)
Second-line therapy proportion, chemotherapy^a^					
Chemotherapy	29.7%	23.8%	35.6%	Beta (173,410)	(6)
Immunotherapy	40.8%	32.6%	49.0%	Beta (238,345)	(6)
Targeted therapy	5.8%	4.6%	7.0%	Beta (34,549)	(6)
Health utility					
N+I with/without chemotherapy	0.88	0.79	0.97	Beta (11.0,1.5)	(30, 31)
Chemotherapy	0.79	0.71	0.87	Beta (20.2,5.4)	(30)
Progressive disease	0.72	0.65	0.79	Beta (27.3,10.6)	(30)
Administration cost ($)^b^	149	120	179	Gamma (100,1.49)	(23)
Drug cost per 6 weeks ($)^b^					
Nivolumab plus ipilimumab	29,890	23,912	35,868	Gamma (100,298.90)	(23, 24)
Gemcitabine plus carboplatin	2528	2022	3034	Gamma (100,25.28)	(23)
Paclitaxel plus carboplatin	502	401	602	Gamma (100,5.02)	(23)
Pemetrexed plus carboplatin	13,791	11,033	16,550	Gamma (100,137.91)	(23)
Docetaxel	834	667	1001	Gamma (100,8.34)	(26)
Nivolumab	18,756	15,005	22,507	Gamma (100,187.56)	(23, 24)
Erlotinib	14,350	11,480	17,220	Gamma (100,143.50)	(25)
Grade 3/4 AEs cost ($)^b^					
Diarrhea	17,668	14,135	21,202	Gamma (100,176.68)	(11, 27)
Rash	16,811	13,449	20,173	Gamma (100,168.11)	(11, 27)
Fatigue	17,320	13,856	20,784	Gamma (100,173.20)	(11, 27)
Decrease appetite	24,814	19,851	29,776	Gamma (100,248.14)	(11, 27)
Nausea	20,698	16,558	24,837	Gamma (100,206.98)	(11, 27)
Anemia	21,681	17,345	26,017	Gamma (100,216.81)	(11, 27)
Neutropenia	18,386	14,709	22,063	Gamma (100,183.86)	(11, 27)
BSC cost per 6 weeks ($)^b^	4894	3915	5873	Gamma (100,48.94)	(28)

a Parameter values for patients with PD-L1 ≥ 1% and < 1% and sensitivity analyses based on the CheckMate 9LA trial are shown in **Supplementary Table 3**.

b All costs are expressed in 2020 dollars.

AE, adverse event; BSC, best supportive care; N+I, nivolumab plus ipilimumab; PD-L1, programmed-death ligand 1; PFS, progression-free survival; OS, overall survival.

## Results

### Base-Case Results

**Figure 1:**
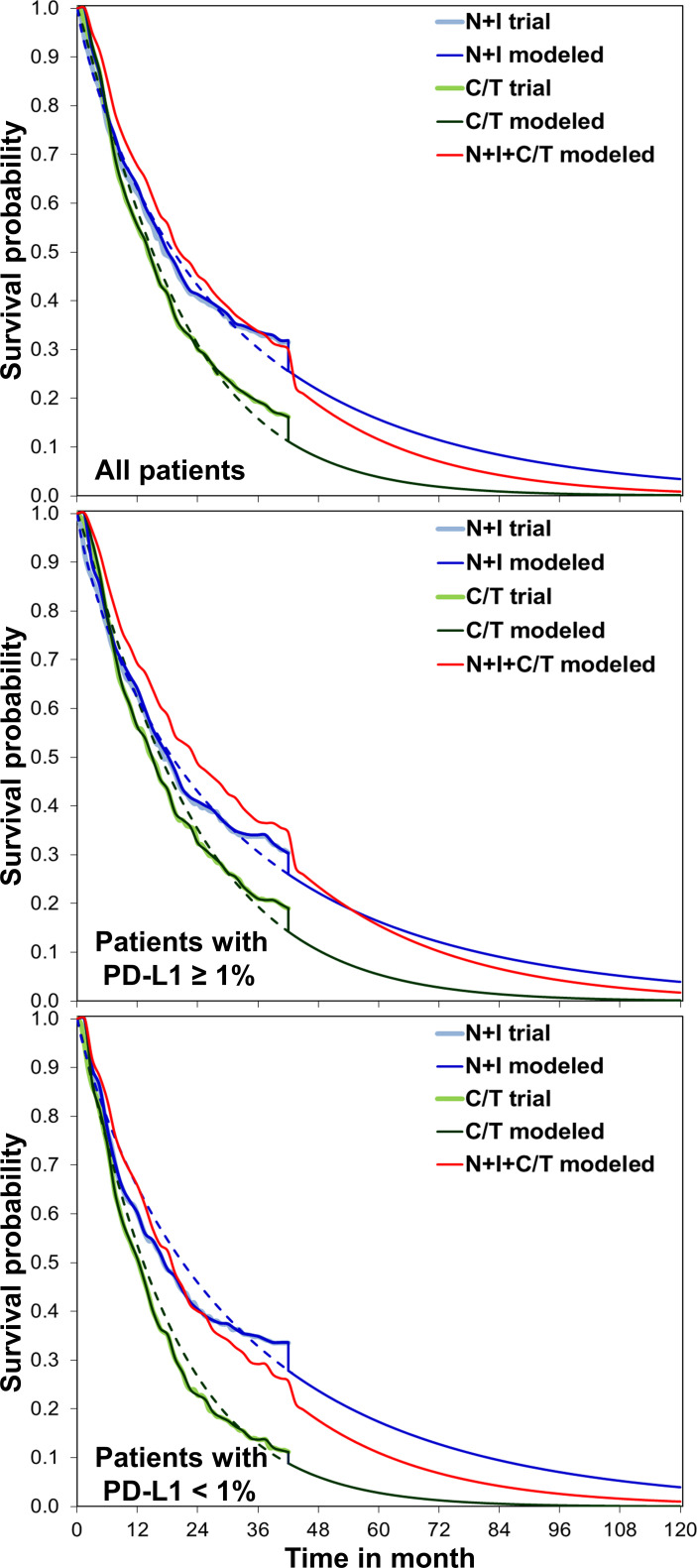
Comparisons between the CheckMate 227 trial and simulation, overall survival for nivolumab plus ipilimumab with two cycles of chemotherapy, nivolumab plus ipilimumab, and chemotherapy. We extrapolated the overall survival to lifetime for analyses. C/T, chemotherapy; N+I, nivolumab plus ipilimumab; PD-L1, programmed-death ligand 1.

The modeled overall survival curves were similar to the results of CheckMate 227 trial, indicating our model was well calibrated (**Figure 1**). **Table 2** shows the base-case results: the mean life expectancies of all patients receiving chemotherapy, N+I, and N+I+chemotherapy were 1.68, 2.16, and 2.35 life years, respectively. After accounting for the quality-of-life weights, the quality-adjusted life expectancies were 1.26, 1.72, and 1.85 QALYs, respectively. The incremental cost for N+I group compared to chemotherapy group was $110,333, and $107,487 for N+I+chemotherapy group compared to N+I group. As a result, the ICER of N+I versus chemotherapy was $228,100 per life year and $239,072 per QALY, and the ICER of N+I+chemotherapy versus N+I was $595,568 per life year and $838,198 per QALY. The respective ICERs for patients with PD-L1 ≥ 1% were $246,584 per QALY and $1,092,784 per QALY. N+I+chemotherapy was dominated by N+I in patients with PD-L1 < 1%.

**Table 2:** Base-case results.

	Total cost	Life years	QALYs	ICER ($/life year)	ICER ($/QALY)
All patients					
Chemotherapy	$175,668	1.68	1.26	–	–
N+I	$286,001	2.16	1.72	228,100	239,072
N+I+chemotherapy	$393,488	2.35	1.85	595,568	838,198
Patients with PD-L1 ≥ 1%					
Chemotherapy	$181,757	1.81	1.35	–	–
N+I	$335,145	2.45	1.98	238,154	246,584
N+I+chemotherapy	$408,078	2.59	2.04	500,896	1,092,784
Patients with PD-L1 < 1%					
Chemotherapy	$151,861	1.37	1.03	–	–
N+I	$338,905	2.59	2.03	152,565	185,620
N+I+chemotherapy	$361,944	2.14	1.67	dominated	dominated

ICER, incremental cost-effectiveness ratio; N+I, nivolumab plus ipilimumab; PD-L1, programmed death-ligand 1; QALY, quality-adjusted life year.

### Sensitivity Analyses

**Figure 2:**
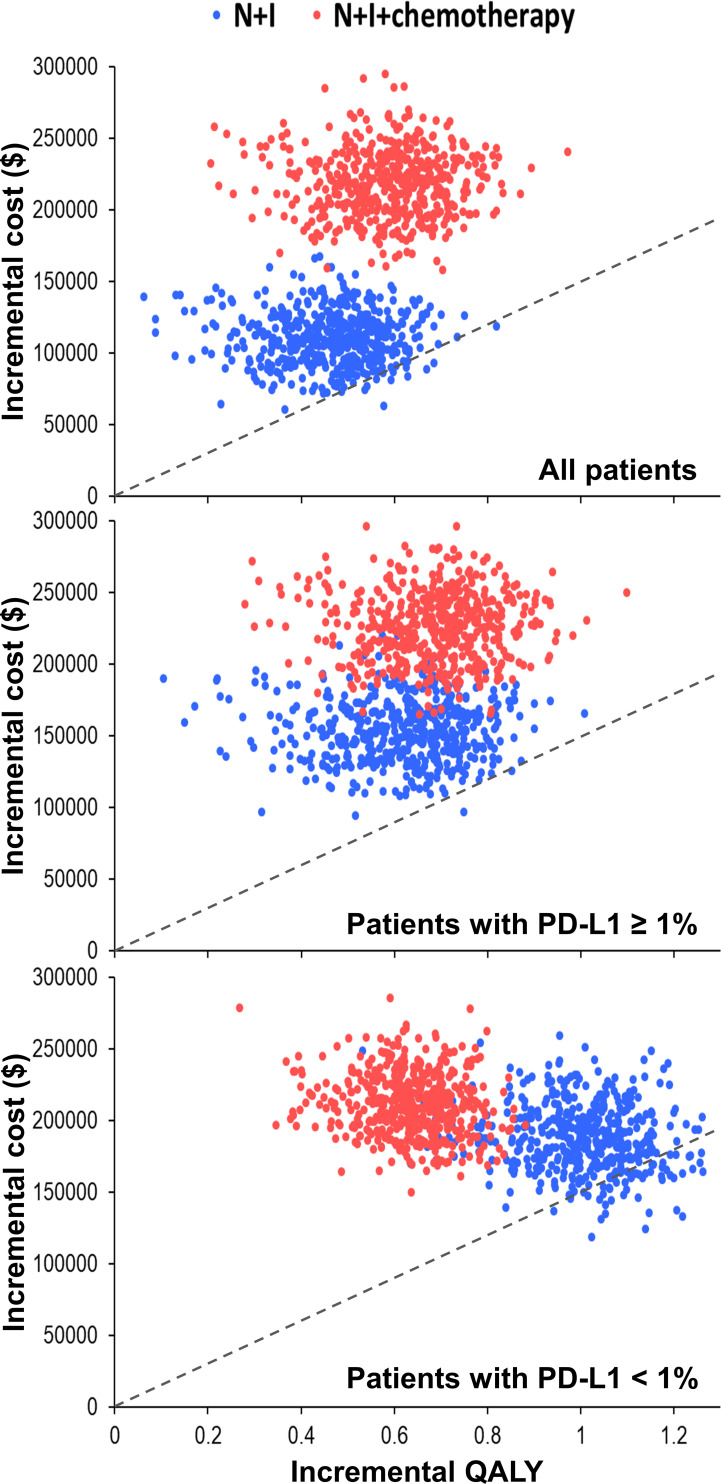
Incremental cost-effectiveness scattered plots using simulation based on the CheckMate 227 trial. Dash lines represent the incremental cost-effectiveness ratio of $150,000 per QALY. N+I, nivolumab plus ipilimumab; PD-L1, programmed-death ligand 1; QALY, quality-adjusted life year.

**Figure 3:**
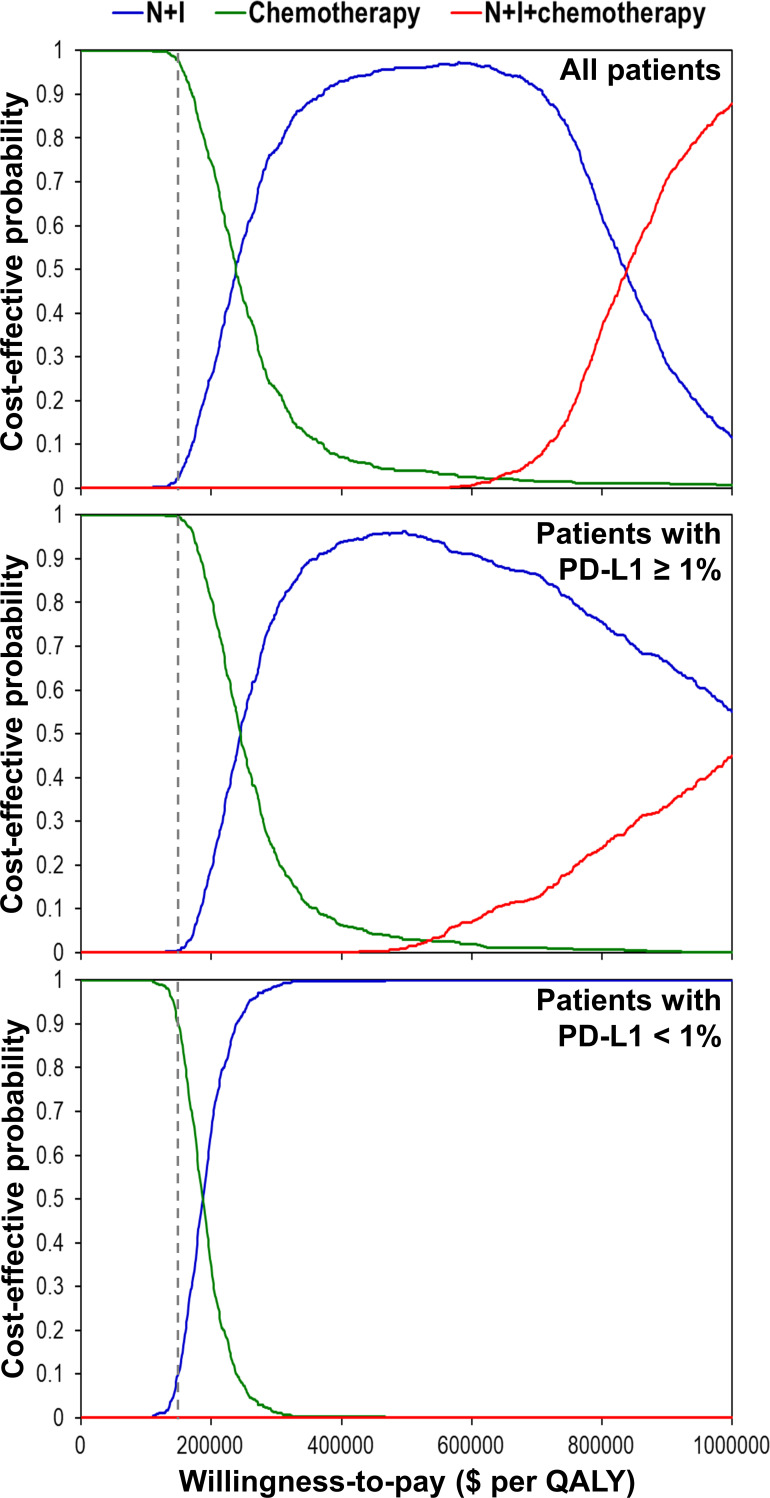
Cost-effectiveness acceptability curves using simulation based on the CheckMate 227 trial. Dash lines represent the willingness-to-pay threshold of $150,000 per QALY. N+I, nivolumab plus ipilimumab; PD-L1, programmed-death ligand 1; QALY, quality-adjusted life year.

One-way deterministic sensitivity analyses (**Supplementary Figure 2**) revealed that the ICER was higher than $150,000 per QALY when we changed each individual estimate within its plausible range except the cost of nivolumab plus ipilimumab. If cost of nivolumab plus ipilimumab reduced to $23,912, the ICER would be $182,253 per QALY for patients with PD-L1 ≥ 1% and $145,802 per QALY for patients with PD-L1 < 1%. Incremental cost-effectiveness scattered plots (**Figure 2**) show that N+I, as compared with chemotherapy, had a 2.6% probability of being cost-effective by falling to the right of dash lines, which represented $150,000 per QALY. Furthermore, N+I+chemotherapy was dominated by N+I for patients with PD-L1 < 1%. Cost-effectiveness acceptability curves (**Figure 3**) reveal that compared with chemotherapy, N+I had a higher probability of being cost-effective at a willingness-to-pay threshold above $238,000 per QALY for all patients, $245,000 per QALY for patients with PD-L1 ≥ 1%, and $187,000 per QALY for patients with PD-L1<1%. N+I+chemotherapy, as compared with N+I, had a higher probability of being cost-effective at a threshold above $838,000 per QALY for all patients.

Sensitivity analyses using the chemotherapy group in CheckMate 9LA trial as the reference (**Supplementary Table 4** and **Figure 3**) show that neither N+I nor N+I+chemotherapy in any scenario could be a cost-effective strategy given a willingness-to-pay threshold of $150,000 per QALY.

## Discussion

This study examined the cost-effectiveness of N+I and N+I+chemotherapy for patients with metastatic NSCLC. We concomitantly compared the two front-line treatments and chemotherapy by abstracting the efficacy and safety data from phase 3 randomized trials (6, 7), and incorporated time-dependent transitional probabilities during the follow-up period for disease progression; thus, our simulation model was able to accurately reflect the effectiveness estimates. Further, instead of simply assigning docetaxel as the second-line chemotherapy for all groups (17), we explicitly modeled the subsequent treatment based on the trial data. We found that first-line N+I and N+I+chemotherapy for metastatic NSCLC were not cost-effective from the U.S. health care sector perspective. Subgroup analyses by different tumor PD-L1 expression levels, and a variety of sensitivity analyses further verified the robustness of the results. Our results provide important information and could help clinical guidelines development when medical community moves toward value-based practice.

Unlike previous investigations which found the ICERs of single-agent ICI regimens versus chemotherapy to be less than $150,000 per QALY (11–15), the ICERs of N+I and N+I+chemotherapy versus chemotherapy alone in our study were far above the willingness-to-pay threshold. Although this finding might be attributed to a higher cost of concomitant use of two ICIs, the ICERs were still higher than the results of three recently published studies (16, 17, 19). One plausible explanation is that we did not include the costs of pemetrexed maintenance therapy after 4 treatment cycles of pemetrexed plus carboplatin chemotherapy, leading to lower cost estimates in our platinum-doublet chemotherapy group. However, pemetrexed maintenance therapy was optional in the trials and could be administered both in the progression-free state of chemotherapy and progressive disease of N+I or N+I+chemotherapy. If we took this issue into consideration and assumed that every non-squamous NSCLC patient received pemetrexed maintenance therapy after pemetrexed plus carboplatin, the ICERs of N+I versus chemotherapy for all patients, patients with PD-L1 ≥ 1%, and patients with PD-L1 < 1% would become $212,823, $220,670, and $200,814 per QALY, respectively, which were still higher than the willingness-to-pay threshold value. In addition, we applied carboplatin plus gemcitabine or pemetrexed as the second-line chemotherapy following progression on N+I+chemotherapy, which would overestimate the ICER of N+I+chemotherapy versus N+I. If we selected docetaxel as the regimen, the ICERs of N+I+chemotherapy versus N+I for all patients and patients with PD-L1 ≥ 1% would become $768,107 and $966,114 per QALY, respectively, indicating N+I+chemotherapy was still not cost-effective. Also, N+I+chemotherapy remained dominated by N+I in patients with PD-L1 < 1%.

As the movement toward value-based practice, our comparison between N+I+chemotherapy and N+I has important clinical implication. Given the increases in health care spending over time, we acknowledge that the willingness-to-pay threshold of $150,000 per QALY might be under-estimated. While researchers have inferred a higher willingness-to-pay threshold, such as $300,000 per QALY (32), our results indicated that adding chemotherapy over N+I is definitely not cost-effective, and should be discouraged.

In contrast to our intuition, the ICER of N+I versus chemotherapy in patients with PD-L1 ≥ 1% was higher than that in patients with PD-L1 < 1%. This finding could be attributed to a lower survival benefit of N+I versus chemotherapy in PD-L1 ≥ 1% subgroup than that in PD-L1 < 1% subgroup (6). Similar ICER results were also recognized in two previous studies (17, 18). Although exploratory analysis of the trial showed that N+I provided preferable survival benefits in patients with PD-L1 ≥ 50% or high tumor mutational burden, we did not perform these subgroup analyses because of the absence of valid data.

We conducted sensitivity analyses using the chemotherapy group in CheckMate 9LA trial as the reference. In contrast to the base-case results on the CheckMate 227 trial, N+I was weakly dominated by N+I+chemotherapy in all patients and patients with PD-L1 ≥ 1%, and N+I no longer dominated N+I+chemotherapy in patient with PD-L1 < 1%. Possible reasons include that simulations based on the CheckMate 9LA trial modeled a better overall survival of N+I+chemotherapy group than that of N+I group. Nevertheless, either N+I or N+I+chemotherapy remained not cost-effective given a willingness-to-pay threshold of $150,000 per QALY.

Several limitations must be acknowledged in our study. First, we assumed no background mortality and every simulated patient transited from progression-free state to progressive disease before death, which might not capture the deaths resulted from other comorbidities. However, the proportion of deaths attributable to other comorbidities in patients with metastatic NSCLC was only about 6% (33), and our modeled overall survival was similar to the trial results. Second, because the health utility data for all patients in the trial were not available, we used the utility difference in a subgroup of patients with high tumor mutational burden (31) to derive the health utility value for patients receiving N+I or N+I+chemotherapy. Although the effect of different adverse events between treatment groups had been accounted for, patients with high tumor mutational burden were supposed to have a better tumor response to ICIs than those with low tumor mutational burden (34). Consequently, the health utility and quality-adjusted life expectancy in the N+I and N+I+chemotherapy groups might be overestimated and the ICERs be underestimated. Nevertheless, wide variabilities of health utility value were examined in the sensitivity analyses, N+I and N+I+chemotherapy remained not cost-effective. Third, we assumed all treatments were discontinued upon disease progression, whereas nivolumab plus ipilimumab could be used beyond disease progression (6, 7), the costs incurred in N+I and N+I+chemotherapy would thus be underestimated, resulting in lower ICER values. Moreover, we did not account for the waste of drugs while calculating their costs. However, more costs would be applied to N+I and N+I+chemotherapy after addressing these issues, further indicating that N+I and N+I+chemotherapy were not cost-effective.

In conclusion, from the U.S. health care sector perspective, first-line N+I or N+I+chemotherapy was not cost-effective for patients with metastatic NSCLC regardless of tumor PD-L1 expressions levels at a willingness-to-pay threshold of $150,000 per QALY.

## Data Availability Statement

Publicly available datasets were analyzed in this study. This data can be found here: N Engl J Med (2016) 375(19):1823-33; N Engl J Med (2018) 378(22):2078-92.

## Ethics Statement

Ethical review and approval was not required for the study on human participants in accordance with the local legislation and institutional requirements. Written informed consent for participation was not required for this study in accordance with the national legislation and the institutional requirements.

## Author Contributions

S-CY had full access to all the data in the study and takes responsibility for the integrity of the data and the accuracy of the data analysis. *Study concept and design:* S-CY and S-YW. *Acquisition, analysis, or interpretation of data:* S-CY. *Drafting of the manuscript:* S-CY and S-YW. *Critical revision of the manuscript for important intellectual content:* All authors. *Statistical analysis:* S-CY and NK. *Obtained funding:* S-CY. *Administrative, technical, or material support:* NK. *Supervision:* CG and S-YW. All authors contributed to the article and approved the submitted version.

## Funding

This work was supported by the Ministry of Science and Technology (110-2314-B-006-100-MY2) and National Cheng Kung University Hospital (NCKUH-11002029). The funder had no role in the design and conduct of the study; collection, management, analysis, and interpretation of the data; preparation, review, or approval of the manuscript; and decision to submit the manuscript for publication.

## Conflict of Interest

S-CY reports grants from the Ministry of Science and Technology and National Cheng University Hospital during the conduct of the study. S-YW reports grants from Genentech outside the submitted work.

The remaining authors declare that the research was conducted in the absence of any commercial or financial relationships that could be construed as a potential conflict of interest.

## Publisher’s Note

All claims expressed in this article are solely those of the authors and do not necessarily represent those of their affiliated organizations, or those of the publisher, the editors and the reviewers. Any product that may be evaluated in this article, or claim that may be made by its manufacturer, is not guaranteed or endorsed by the publisher.

## References

[1] Reck M Rodriguez-Abreu D Robinson AG Hui R Csoszi T Fulop A. Pembrolizumab Versus Chemotherapy for PD-L1-Positive Non-Small-Cell Lung Cancer. N Engl J Med (2016) 375(19):1823–33. doi: 10.1056/NEJMoa1606774 27718847

[2] Gandhi L Rodríguez-Abreu D Gadgeel S Esteban E Felip E De Angelis F. Pembrolizumab Plus Chemotherapy in Metastatic non–Small-Cell Lung Cancer. N Engl J Med (2018) 378(22):2078–92. doi: 10.1056/NEJMoa1801005 29658856

[3] Paz-Ares L Luft A Vicente D Tafreshi A Gümüş M Mazières J. Pembrolizumab Plus Chemotherapy for Squamous Non-Small-Cell Lung Cancer. N Engl J Med (2018) 379(21):2040–51. doi: 10.1056/NEJMoa1810865 30280635

[4] West H McCleod M Hussein M Morabito A Rittmeyer A Conter HJ. Atezolizumab in Combination With Carboplatin Plus Nab-Paclitaxel Chemotherapy Compared With Chemotherapy Alone as First-Line Treatment for Metastatic Non-Squamous Non-Small-Cell Lung Cancer (IMpower130): A Multicentre, Randomised, Open-Label, Phase 3 Trial. Lancet Oncol (2019) 20(7):924–37. doi: 10.1016/S1470-2045(19)30167-6 31122901

[5] Socinski MA Jotte RM Cappuzzo F Orlandi F Stroyakovskiy D Nogami N. Atezolizumab for First-Line Treatment of Metastatic Nonsquamous NSCLC. N Engl J Med (2018) 378(24):2288–301. doi: 10.1056/NEJMoa1716948 29863955

[6] Hellmann MD Paz-Ares L Bernabe Caro R Zurawski B Kim SW Carcereny Costa E. Nivolumab Plus Ipilimumab in Advanced Non-Small-Cell Lung Cancer. N Engl J Med (2019) 381(21):2020–31. doi: 10.1056/NEJMoa1910231 31562796

[7] Paz-Ares L Ciuleanu TE Cobo M Schenker M Zurawski B Menezes J. First-Line Nivolumab Plus Ipilimumab Combined With Two Cycles of Chemotherapy in Patients With Non-Small-Cell Lung Cancer (CheckMate 9LA): An International, Randomised, Open-Label, Phase 3 Trial. Lancet Oncol (2021) 22(2):198–211. doi: 10.1016/S1470-2045(20)30641-0 33476593

[8] U.S. Food & Drug Administration. FDA Approves Nivolumab Plus Ipilimumab for First-Line mNSCLC (PD-L1 Tumor Expression ≥1%). Available at: https://www.fda.gov/drugs/drug-approvals-and-databases/fda-approves-nivolumab-plus-ipilimumab-first-line-mnsclc-pd-l1-tumor-expression-1 (Accessed 13 June 2021).

[9] National Comprehensive Cancer Network. Non-Small Cell Lung Cancer (Version 4.2021). Available at: https://www.nccn.org/professionals/physician_gls/pdf/nscl.pdf (Accessed 9 March 2021).

[10] U.S. Food & Drug Administration. FDA Approves Nivolumab Plus Ipilimumab and Chemotherapy for First-Line Treatment of Metastatic NSCLC. Available at: https://www.fda.gov/drugs/resources-information-approved-drugs/fda-approves-nivolumab-plus-ipilimumab-and-chemotherapy-first-line-treatment-metastatic-nsclc (Accessed 13 June 2021).

[11] Georgieva M da Silveira Nogueira Lima JP Aguiar P Jr. de Lima Lopes G Jr. Haaland B. Cost-Effectiveness of Pembrolizumab as First-Line Therapy for Advanced Non-Small Cell Lung Cancer. Lung Cancer (2018) 124:248–54. doi: 10.1016/j.lungcan.2018.08.018 30268469

[12] Huang M Lou Y Pellissier J Burke T Liu FX Xu R. Cost Effectiveness of Pembrolizumab vs. Standard-of-Care Chemotherapy as First-Line Treatment for Metastatic NSCLC That Expresses High Levels of PD-L1 in the United States. PharmacoEconomics (2017) 35(8):831–44. doi: 10.1007/s40273-017-0527-z PMC5548835 28620848

[13] Huang M Lopes GL Insinga RP Burke T Ejzykowicz F Zhang Y. Cost-Effectiveness of Pembrolizumab Versus Chemotherapy as First-Line Treatment in PD-L1-Positive Advanced Non-Small-Cell Lung Cancer in the USA. Immunotherapy (2019) 11(17):1463–78. doi: 10.2217/imt-2019-0178 31738117

[14] Insinga RP Vanness DJ Feliciano JL Vandormael K Traore S Burke T. Cost-Effectiveness of Pembrolizumab in Combination With Chemotherapy in the 1st Line Treatment of Non-Squamous NSCLC in the US. J Med Econ (2018) 21(12):1191–205. doi: 10.1080/13696998.2018.1521416 30188231

[15] Insinga RP Vanness DJ Feliciano JL Vandormael K Traore S Ejzykowicz F. Cost-Effectiveness of Pembrolizumab in Combination With Chemotherapy Versus Chemotherapy and Pembrolizumab Monotherapy in the First-Line Treatment of Squamous Non-Small-Cell Lung Cancer in the US. Curr Med Res Opin (2019) 35(7):1241–56. doi: 10.1080/03007995.2019.1571297 30649973

[16] Hu H She L Liao M Shi Y Yao L Ding D. Cost-Effectiveness Analysis of Nivolumab Plus Ipilimumab vs. Chemotherapy as First-Line Therapy in Advanced Non-Small Cell Lung Cancer. Front Oncol (2020) 10:1649. doi: 10.3389/fonc.2020.01649 33014826 PMC7507990

[17] Li J Zhang T Xu Y Lu P Zhu J Liang W. Cost-Effectiveness Analysis of Nivolumab Plus Ipilimumab Versus Chemotherapy as First-Line Treatment in Advanced NSCLC. Immunotherapy (2020) 12(14):1067–75. doi: 10.2217/imt-2020-0112 32811247

[18] Courtney PT Yip AT Cherry DR Salans MA Kumar A Murphy JD. Cost-Effectiveness of Nivolumab-Ipilimumab Combination Therapy for the Treatment of Advanced Non-Small Cell Lung Cancer. JAMA Netw Open (2021) 4(5):e218787. doi: 10.1001/jamanetworkopen.2021.8787 33938936 PMC8094011

[19] Peng Y Zeng X Peng L Liu Q Yi L Luo X. Cost-Effectiveness of Nivolumab Plus Ipilimumab Combined With Two Cycles of Chemotherapy as First-Line Treatment in Advanced Non-Small Cell Lung Cancer. Adv Ther (2021) 38(7):3962–72. doi: 10.1007/s12325-021-01788-6 34100243

[20] Neumann PJ Cohen JT Weinstein MC. Updating Cost-Effectiveness — The Curious Resilience of the $50,000-Per-QALY Threshold. N Engl J Med (2014) 371(9):796–7. doi: 10.1056/NEJMp1405158 25162885

[21] Sanders GD Neumann PJ Basu A Brock DW Feeny D Krahn M. Recommendations for Conduct, Methodological Practices, and Reporting of Cost-Effectiveness Analyses: Second Panel on Cost-Effectiveness in Health and Medicine. JAMA (2016) 316(10):1093–103. doi: 10.1001/jama.2016.12195 27623463

[22] Hoyle MW Henley W. Improved Curve Fits to Summary Survival Data: Application to Economic Evaluation of Health Technologies. BMC Med Res Methodol (2011) 11:139. doi: 10.1186/1471-2288-11-139 21985358 PMC3198983

[23] She L Hu H Liao M Xia X Shi Y Yao L. Cost-Effectiveness Analysis of Pembrolizumab Versus Chemotherapy as First-Line Treatment in Locally Advanced or Metastatic Non-Small Cell Lung Cancer With PD-L1 Tumor Proportion Score 1% or Greater. Lung Cancer (2019) 138:88–94. doi: 10.1016/j.lungcan.2019.10.017 31655368

[24] Wan X Zhang Y Tan C Zeng X Peng L. First-Line Nivolumab Plus Ipilimumab vs Sunitinib for Metastatic Renal Cell Carcinoma: A Cost-Effectiveness Analysis. JAMA Oncol (2019) 5(4):491–6. doi: 10.1001/jamaoncol.2018.7086 PMC6459127 30789633

[25] Aguiar PN Jr Haaland B Park W San Tan P Del Giglio A de Lima Lopes G Jr. Cost-Effectiveness of Osimertinib in the First-Line Treatment of Patients With EGFR-Mutated Advanced Non-Small Cell Lung Cancer. JAMA Oncol (2018) 4(8):1080–4. doi: 10.1001/jamaoncol.2018.1395 PMC6143050 29852038

[26] Wan X Luo X Tan C Zeng X Zhang Y Peng L. First-Line Atezolizumab in Addition to Bevacizumab Plus Chemotherapy for Metastatic, Nonsquamous Non-Small Cell Lung Cancer: A United States-Based Cost-Effectiveness Analysis. Cancer (2019) 125(20):3526–34. doi: 10.1002/cncr.32368 31287562

[27] Wong W Yim YM Kim A Cloutier M Gauthier-Loiselle M Gagnon-Sanschagrin P. Assessment of Costs Associated With Adverse Events in Patients With Cancer. PLoS One (2018) 13(4):e0196007. doi: 10.1371/journal.pone.0196007 29652926 PMC5898735

[28] Criss SD Mooradian MJ Sheehan DF Zubiri L Lumish MA Gainor JF. Cost-Effectiveness and Budgetary Consequence Analysis of Durvalumab Consolidation Therapy vs No Consolidation Therapy After Chemoradiotherapy in Stage III Non-Small Cell Lung Cancer in the Context of the US Health Care System. JAMA Oncol (2019) 5(3):358–65. doi: 10.1001/jamaoncol.2018.5449 PMC6439842 30543349

[29] U.S. Bureau of Labor Statistics. Consumer Price Index - CPI Latest Numbers. Available at: https://www.bls.gov/cpi/latest-numbers.htm (Accessed 22 June 2021).

[30] Yang SC Kuo CW Lai WW Lin CC Su WC Chang SM. Dynamic Changes of Health Utility in Lung Cancer Patients Receiving Different Treatments: A 7-Year Follow-Up. J Thorac Oncol (2019) 14(11):1892–900. doi: 10.1016/j.jtho.2019.07.007 31352073

[31] Reck M Schenker M Lee KH Provencio M Nishio M Lesniewski-Kmak K. Nivolumab Plus Ipilimumab Versus Chemotherapy as First-Line Treatment in Advanced Non-Small-Cell Lung Cancer With High Tumour Mutational Burden: Patient-Reported Outcomes Results From the Randomised, Open-Label, Phase III CheckMate 227 Trial. Eur J Cancer (2019) 116:137–47. doi: 10.1016/j.ejca.2019.05.008 31195357

[32] Braithwaite RS Meltzer DO King JT Jr. Leslie D Roberts MS. What Does the Value of Modern Medicine Say About the $50,000 Per Quality-Adjusted Life-Year Decision Rule? Med Care (2008) 46(4):349–56. doi: 10.1097/MLR.0b013e31815c31a7 18362813

[33] Tan KS Eguchi T Adusumilli PS. Reporting Net Survival in Populations: A Sensitivity Analysis in Lung Cancer Demonstrates the Differential Implications of Reporting Relative Survival and Cause-Specific Survival. Clin Epidemiol (2019) 11:781–92. doi: 10.2147/CLEP.S210894 PMC6730547 31564983

[34] Hellmann MD Ciuleanu TE Pluzanski A Lee JS Otterson GA Audigier-Valette C. Nivolumab Plus Ipilimumab in Lung Cancer With a High Tumor Mutational Burden. N Engl J Med (2018) 378(22):2093–104. doi: 10.1056/NEJMoa1801946 PMC7193684 29658845

