# Supplementary material for: Cost-Effectiveness of Nivolumab Plus Ipilimumab With and Without Chemotherapy for Advanced Non-Small Cell Lung Cancer
Source: Front Oncol. 2021 Dec 9;11:760686. doi: 10.3389/fonc.2021.760686 (PMC8695441; doi:10.3389/fonc.2021.760686)
Supplement: Supplementary file 1 [file DataSheet_1.doc]

Supplementary material

**Cost-effectiveness of nivolumab plus ipilimumab with and without chemotherapy for advanced non-small cell lung cancer**

Szu-Chun Yang, Natalia Kunst, Cary P. Gross, Jung-Der Wang, Wu-Chou Su and Shi-Yi Wang*

*Correspondence: Shi-Yi Wang, MD, PhD, Yale School of Public Health, 60 College Street, New Haven, CT 06510, USA. E-mail: shiyi.wang@yale.edu; telephone: 203.737.8096

**Supplementary Figure 1** Model structure. NSCLC, non-small cell lung cancer.

**Supplementary Figure 2** One-way sensitivity analyses. The bold dash lines represent the ICERs of N+I versus chemotherapy and N+I+chemotherapy versus N+I for all patients, patients with PD-L1 ≥ 1%, and patients with PD-L1 < 1%. BSC, best supportive care; C/T, chemotherapy; ICER, incremental cost-effectiveness ratio; IO, immuno-oncology agent; N+I, nivolumab plus ipilimumab; PD, progressive disease; PD-L1, programmed-death ligand 1; QALY, quality-adjusted life year.


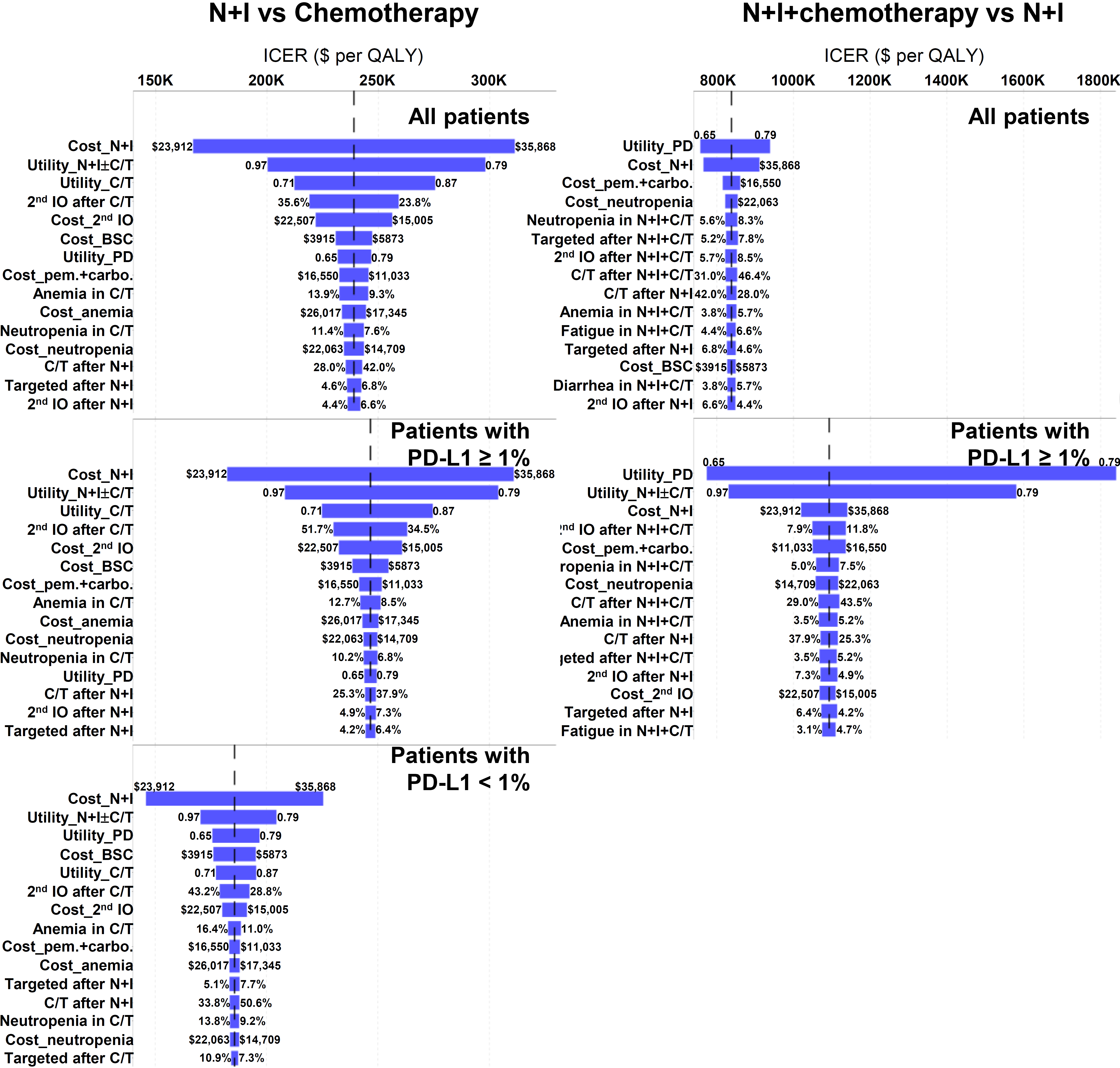


**Supplementary Figure 3** Sensitivity analyses base on the CheckMate 9LA trial. C/T, chemotherapy; N+I, nivolumab plus ipilimumab; PD-L1, programmed-death ligand 1; QALY, quality-adjusted life year.


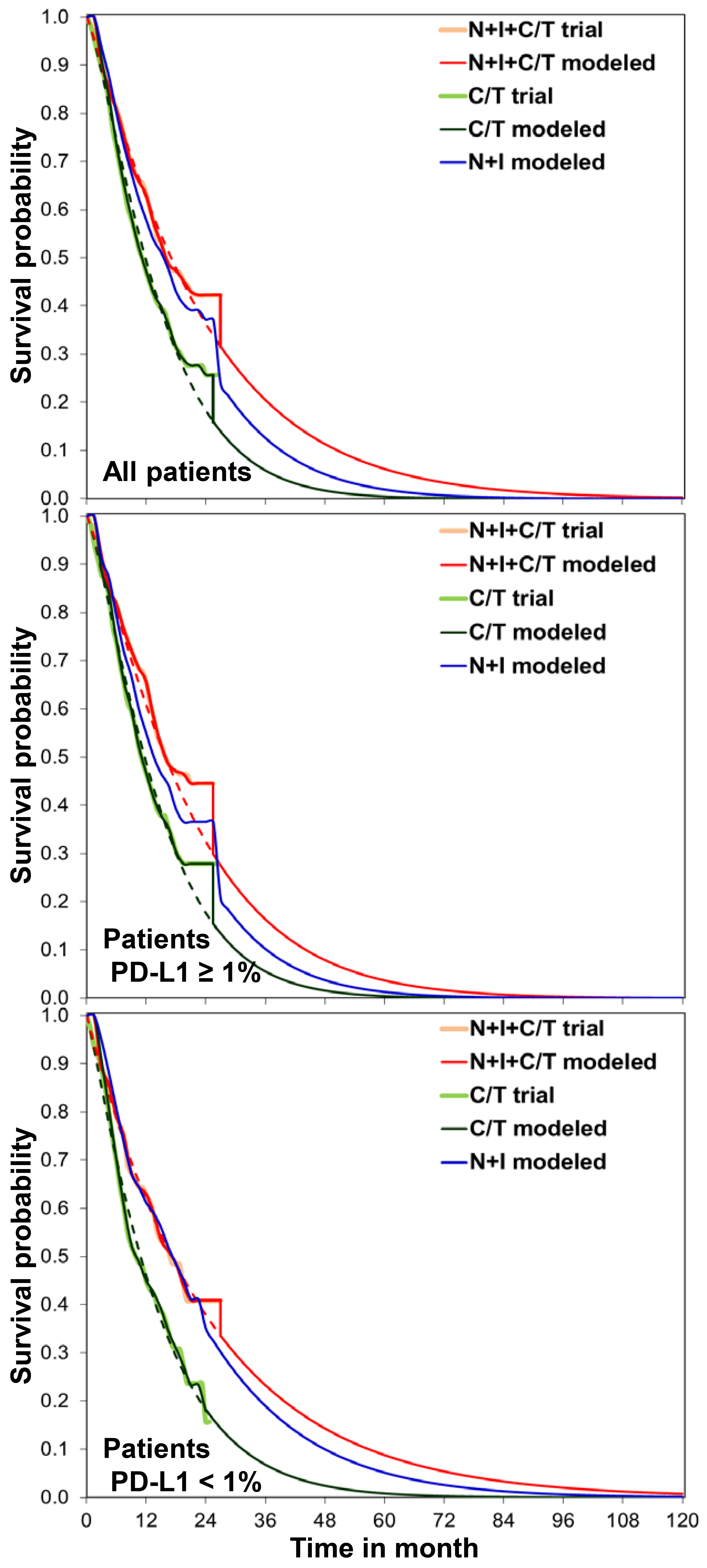


| **Supplementary Table 1** Subsequent treatments in the CheckMate 227 and 9LA trials | | | | | | | | | | | | |
| --- | --- | --- | --- | --- | --- | --- | --- | --- | --- | --- | --- | --- |
|  | CheckMate 227 (1) | | | | | | CheckMate 9LA (2) | | | | | |
|  | All patients | | Patients with  PD-L1 ≥ 1% | | Patients with  PD-L1 < 1% | | All patients | | Patients with  PD-L1 ≥ 1% | | Patients with  PD-L1 < 1% | |
|  | N+I  *n*=583 | C/T  *n*=583 | N+I  *n*=396 | C/T  *n*=397 | N+I  *n*=187 | C/T  *n*=186 | N+I+C/T  *n*=361 | C/T  *n*=358 | N+I+C/T  *n*=203 | C/T  *n*=204 | N+I+C/T  *n*=135 | C/T  *n*=129 |
| Chemotherapy | 204  (35.0%) | 173  (29.7%) | 125  (31.6%) | 109  (27.5%) | 79  (42.2%) | 64  (34.4%) | 105  (29.1%) | 80  (22.3%) | 59  (29.1%) | 45  (22.1%) | 42  (31.1%) | 32  (24.8%) |
| Immunotherapy | 32  (5.5%) | 238  (40.8%) | 24  (6.1%) | 171  (43.1%) | 8  (4.3%) | 67  (36.0%) | 19  (5.3%) | 108  (30.2%) | 15  (7.4%) | 66  (32.4%) | 4  (3.0%) | 35  (27.1%) |
| Nivolumab | 20  (3.4%) | 185  (31.7%) | 16  (4.0%) | 129  (32.5%) | 4  (2.1%) | 56  (30.1%) | 5  (1.4%) | 56  (15.6%) | 5  (2.5%) | 32  (15.7%) | 0 | 21  (16.3%) |
| Pembrolizumab | 8  (1.4%) | 38  (6.5%) | 5  (1.3%) | 32  (8.1%) | 3  (1.6%) | 6  (3.2%) | 3  (0.8%) | 31  (8.7%) | 2  (1.0%) | 21  (10.3%) | 1  (0.7%) | 6  (4.7%) |
| Atezolizumab | 3  (0.5%) | 9  (1.5%) | 2  (0.5%) | 5  (1.3%) | 1  (0.5%) | 4  (2.2%) | 4  (1.1%) | 17  (4.7%) | 3  (1.5%) | 11  (5.4%) | 1  (0.7%) | 6  (4.7%) |
| Targeted therapy | 33  (5.7%) | 34  (5.8%) | 21  (5.3%) | 17  (4.3%) | 12  (6.4%) | 17  (9.1%) | 18  (5.0%) | 16  (4.5%) | 9  (4.4%) | 9  (4.4%) | 9  (6.7%) | 7  (5.4%) |
| C/T, chemotherapy; N+I, nivolumab plus ipilimumab; PD-L1, programmed death-ligand 1. | | | | | | | | | | | | |

**References**

1. Hellmann MD, Paz-Ares L, Bernabe Caro R, Zurawski B, Kim SW, Carcereny Costa E, et al. Nivolumab plus ipilimumab in advanced non-small-cell lung cancer. *New Engl J Med* (2019) 381(21):2020-31.

2. Paz-Ares L, Ciuleanu TE, Cobo M, Schenker M, Zurawski B, Menezes J, et al. First-line nivolumab plus ipilimumab combined with two cycles of chemotherapy in patients with non-small-cell lung cancer (CheckMate 9LA): an international, randomised, open-label, phase 3 trial. *Lancet Oncol* (2021) 22(2):198-211.

| **Supplementary Table 2** Doses and costs of drugs | | | |
| --- | --- | --- | --- |
| Drug | Dose | Unit price | Cost per 6 weeks |
| Nivolumab | 3 mg/kg * 70 kg every 2 weeks up to 2 years | $29 per 1 mg | $18,308 |
| Ipilimumab | 1 mg/kg * 70 kg every 6 weeks up to 2 years | $159 per 1 mg | $11,134 |
| Gemcitabine | 1000 mg/m^2^ * 1.84 m^2^, day 1 and 8, every 3 weeks up to 12 weeks | $25 per 100 mg | $1849 |
| Paclitaxel | 200 mg * 1.84 m^2^ every 3 weeks up to 12 weeks | $16 per 100mg | $121 |
| Pemetrexed | 500 mg/m^2^ * 1.84 m^2^ every 3 weeks up to 12 weeks | $729 per 100 mg | $13,411 |
| Carboplatin | 587.5 mg (AUC: 6 mg/ml/min) every 3 weeks up to 12 weeks | $7.0 per 100 mg | $82 |
| Erlotinib | 150 mg per day | $342 per 150 mg | $14,350 |
| Docetaxel | 75 mg/m^2^ *1.84 m^2^ every 3 weeks up to 12 weeks | $194 per 100 mg | $535 |
| AUC, area under the concentration-time curve. | | | |

| **Supplementary Table 3** Parameter values for patients with PD-L1 ≥ 1% and < 1% and sensitivity analyses based on the CheckMate 9LA trial | | | | |
| --- | --- | --- | --- | --- |
| Parameter | Base-case analyses based on the  CheckMate 227 | | Sensitivity analyses based on the  CheckMate 9LA | |
|  | Baseline value | Distribution | Baseline value | Distribution |
| Transitional probabilities | Estimated from the trial and extrapolated survival curves(1) |  | Estimated from the trial and extrapolated survival curves (2) |  |
| Hazard ratios of N+I+chemotherapy versus chemotherapy | | | | |
| PFS, patients with PD-L1 ≥ 1% | 0.67(2) |  |  |  |
| PFS, patients with PD-L1 < 1% | 0.71(2) |  |  |  |
| OS, patients with PD-L1 ≥ 1% | 0.64(2) |  |  |  |
| OS, patients with PD-L1 < 1% | 0.62(2) |  |  |  |
| Hazard ratios of N+I versus chemotherapy | | | | |
| PFS, all patients |  |  | 0.79(1) |  |
| PFS, patients with PD-L1 ≥ 1% |  |  | 0.82(1) |  |
| PFS, patients with PD-L1 < 1% |  |  | 0.75(1) |  |
| OS, all patients |  |  | 0.73(1) |  |
| OS, patients with PD-L1 ≥ 1% |  |  | 0.79(1) |  |
| OS, patients with PD-L1 < 1% |  |  | 0.62(1) |  |
| Squamous in tumor histology | | | | |
| All patients |  |  | 31.3%(2) | Dirichlet (113,248) |
| Patients with PD-L1 ≥ 1% | 29.5%(1) | Dirichlet (117,279) | 31.3%(2) | Dirichlet (113,248) |
| Patients with PD-L1 < 1% | 24.7%(1) | Dirichlet (46,140) | 31.3%(2) | Dirichlet (113,248) |
| Grade 3/4 AEs incidence for all patients, N+I+chemotherapy | | | | |
| Diarrhea |  |  | 3.9%(2) | Beta (14,344) |
| Rash |  |  | 1.7%(2) | Beta (6,352) |
| Fatigue |  |  | 2.2%(2) | Beta (8,350) |
| Decreased appetite |  |  | 1.1%(2) | Beta (4,354) |
| Nausea |  |  | 1.4%(2) | Beta (5,353) |
| Anemia |  |  | 5.9%(2) | Beta (21,337) |
| Neutropenia |  |  | 6.7%(2) | Beta (24,334) |
| Grade 3/4 AEs incidence for patients with PD-L1 ≥ 1%, N+I+chemotherapy | | | | |
| Diarrhea | 3.4%(1, 2) | Beta (7,196) | 3.9%(2) | Beta (8,195) |
| Rash | 1.7%(1, 2) | Beta (3,200) | 1.7%(2) | Beta (3,200) |
| Fatigue | 3.9%(1, 2) | Beta (8,195) | 2.2%(2) | Beta (5,198) |
| Decreased appetite | 1.0%(1, 2) | Beta (2,201) | 1.1%(2) | Beta (2,201) |
| Nausea | 2.9%(1, 2) | Beta (6,197) | 1.4%(2) | Beta (3,200) |
| Anemia | 4.3%(1, 2) | Beta (9,194) | 5.9%(2) | Beta (12,191) |
| Neutropenia | 6.2%(1, 2) | Beta (13,190) | 6.7%(2) | Beta (14,189) |
| Grade 3/4 AEs incidence for patients with PD-L1 < 1%, N+I+chemotherapy | | | | |
| Diarrhea | 7.5%(1, 2) | Beta (10,125) | 3.9%(2) | Beta (5,130) |
| Rash | 1.7%(1, 2) | Beta (2,133) | 1.7%(2) | Beta (2,133) |
| Fatigue | 8.6%(1, 2) | Beta (12,123) | 2.2%(2) | Beta (3,132) |
| Decreased appetite | 1.6%(1, 2) | Beta (2,133) | 1.1%(2) | Beta (2,133) |
| Nausea | 4.4%(1, 2) | Beta (6,129) | 1.4%(2) | Beta (2,133) |
| Anemia | 5.6%(1, 2) | Beta (8,127) | 5.9%(2) | Beta (8,127) |
| Neutropenia | 8.4%(1, 2) | Beta (11,124) | 6.7%(2) | Beta (9,126) |
| Grade 3/4 AEs incidence for all patients, N+I | | | | |
| Diarrhea |  |  | 1.4%(1, 2) | Beta (8, 568) |
| Rash |  |  | 1.6%(1, 2) | Beta (9,567) |
| Fatigue |  |  | 0.7%(1, 2) | Beta (4,572) |
| Decreased appetite |  |  | 0.7%(1, 2) | Beta (4,572) |
| Nausea |  |  | 0.2%(1, 2) | Beta (1,575) |
| Anemia |  |  | 1.7%(1, 2) | Beta (10,566) |
| Grade 3/4 AEs incidence for patients with PD-L1 ≥ 1%, N+I | | | | |
| Diarrhea | 1.5%(1) | Beta (6,385) | 1.7%(1, 2) | Beta (7,384) |
| Rash | 2.3%(1) | Beta (9,382) | 2.3%(1, 2) | Beta (9,382) |
| Fatigue | 2.0%(1) | Beta (8,383) | 1.2%(1, 2) | Beta (4,387) |
| Decreased appetite | 1.0%(1) | Beta (4,387) | 1.2%(1, 2) | Beta (4,387) |
| Nausea | 0.5%(1) | Beta (2,389) | 0.2%(1, 2) | Beta (1,390) |
| Anemia | 1.3%(1) | Beta (5,386) | 1.8%(1, 2) | Beta (7,384) |
| Grade 3/4 AEs incidence for patients with PD-L1 < 1%, N+I | | | | |
| Diarrhea | 2.2%(1) | Beta (4,181) | 1.2%(1, 2) | Beta (2,183) |
| Fatigue | 1.1%(1) | Beta (2,183) | 0.3%(1, 2) | Beta (1,184) |
| Nausea | 0.5%(1) | Beta (1,184) | 0.2%(1, 2) | Beta (0,185) |
| Anemia | 1.6%(1) | Beta (3,182) | 1.7%(1, 2) | Beta (3,182) |
| Grade 3/4 AEs incidence for all patients, chemotherapy | | | | |
| Diarrhea |  |  | 0.6%(2) | Beta (2,347) |
| Fatigue |  |  | 0.6%(2) | Beta (2,347) |
| Decreased appetite |  |  | 1.2%(2) | Beta (4,345) |
| Nausea |  |  | 0.9%(2) | Beta (3,346) |
| Anemia |  |  | 14.3%(2) | Beta (50,299) |
| Neutropenia |  |  | 9.2%(2) | Beta (32,317) |
| Grade 3/4 AEs incidence for patients with PD-L1 ≥ 1%, chemotherapy | | | | |
| Diarrhea | 0.5%(1) | Beta (2,385) | 0.6%(2) | Beta (1,203) |
| Fatigue | 1.0%(1) | Beta (4,383) | 0.6%(2) | Beta (1,203) |
| Decreased appetite | 1.0%(1) | Beta (4,383) | 1.2%(2) | Beta (2,202) |
| Nausea | 1.8%(1) | Beta (7,380) | 0.9%(2) | Beta (2,202) |
| Anemia | 10.6%(1) | Beta (41,346) | 14.3%(2) | Beta (29,175) |
| Neutropenia | 8.5%(1) | Beta (33,354) | 9.2%(2) | Beta (19,185) |
| Grade 3/4 AEs incidence for patients with PD-L1 < 1%, chemotherapy | | | | |
| Diarrhea | 1.1%(1) | Beta (2,181) | 0.6%(2) | Beta (1,128) |
| Fatigue | 2.2%(1) | Beta (4,179) | 0.6%(2) | Beta (1,128) |
| Decreased appetite | 1.6%(1) | Beta (3,180) | 1.2%(2) | Beta (1,128) |
| Nausea | 2.7%(1) | Beta (5,178) | 0.9%(2) | Beta (1,128) |
| Anemia | 13.7%(1) | Beta (25,158) | 14.3%(2) | Beta (18,111) |
| Neutropenia | 11.5%(1) | Beta (21,162) | 9.2%(2) | Beta (12,117) |
| Second-line therapy proportion for all patients, N+I+chemotherapy | | | | |
| Chemotherapy |  |  | 29.1%(2) | Beta (105,256) |
| Immunotherapy |  |  | 5.3%(2) | Beta (19,342) |
| Targeted therapy |  |  | 5.0%(2) | Beta (18,343) |
| Second-line therapy proportion for patients with PD-L1 ≥ 1%, N+I+chemotherapy | | | | |
| Chemotherapy | 36.2%(1, 2) | Beta (74,129) | 29.1%(2) | Beta (59,144) |
| Immunotherapy | 9.8%(1, 2) | Beta (20,183) | 7.4%(2) | Beta (15,188) |
| Targeted therapy | 4.3%(1, 2) | Beta (9,194) | 4.4%(2) | Beta (9,194) |
| Second-line therapy proportion for patients with PD-L1 < 1%, N+I+chemotherapy | | | | |
| Chemotherapy | 43.1%(1, 2) | Beta (58,77) | 31.1%(2) | Beta (42,93) |
| Immunotherapy | 3.9%(1, 2) | Beta (5,130) | 3.0%(2) | Beta (4,131) |
| Targeted therapy | 11.2%(1, 2) | Beta (15,120) | 6.7%(2) | Beta (9,126) |
| Second-line therapy proportion for all patients, N+I | | | | |
| Chemotherapy |  |  | 26.3%(1, 2) | Beta (154,429) |
| Immunotherapy |  |  | 4.1%(1, 2) | Beta (24,559) |
| Targeted therapy |  |  | 4.4%(1, 2) | Beta (26,557) |
| Second-line therapy proportion for patients with PD-L1 ≥ 1%, N+I | | | | |
| Chemotherapy | 31.6%(1) | Beta (125,271) | 25.4%(1, 2) | Beta (100,296) |
| Immunotherapy | 6.1%(1) | Beta (24,372) | 4.6%(1, 2) | Beta (18,378) |
| Targeted therapy | 5.3%(1) | Beta (21,375) | 5.4%(1, 2) | Beta (22,374) |
| Second-line therapy proportion for patients with PD-L1 < 1%, N+I | | | | |
| Chemotherapy | 42.2%(1) | Beta (79,108) | 30.4%(1, 2) | Beta (57,130) |
| Immunotherapy | 4.3%(1) | Beta (8,179) | 3.2%(1, 2) | Beta (6,181) |
| Targeted therapy | 6.4%(1) | Beta (12,175) | 3.8%(1, 2) | Beta (7,180) |
| Second-line therapy proportion for all patients, chemotherapy | | | | |
| Chemotherapy |  |  | 22.4%(2) | Beta (80,278) |
| Immunotherapy |  |  | 30.2%(2) | Beta (108,250) |
| Targeted therapy |  |  | 4.5%(2) | Beta (16,342) |
| Second-line therapy proportion for patients with PD-L1 ≥ 1%, chemotherapy | | | | |
| Chemotherapy | 27.5%(1) | Beta (109,288) | 22.1%(2) | Beta (45,159) |
| Immunotherapy | 43.1%(1) | Beta (171,226) | 32.4%(2) | Beta (66,138) |
| Targeted therapy | 4.3%(1) | Beta (17,380) | 4.4%(2) | Beta (9,195) |
| Second-line therapy proportion for patients with PD-L1 < 1%, chemotherapy | | | | |
| Chemotherapy | 34.4%(1) | Beta (64,122) | 24.8%(2) | Beta (32,97) |
| Immunotherapy | 36.0%(1) | Beta (67,119) | 27.1%(2) | Beta (35,94) |
| Targeted therapy | 9.1%(1) | Beta (17,169) | 5.4%(2) | Beta (7,122) |
| AE, adverse event; N+I, nivolumab plus ipilimumab; PD-L1, programmed-death ligand 1; PFS, progression-free survival; OS, overall survival. | | | | |

**References**

1. Hellmann MD, Paz-Ares L, Bernabe Caro R, Zurawski B, Kim SW, Carcereny Costa E, et al. Nivolumab plus ipilimumab in advanced non-small-cell lung cancer. *New Engl J Med* (2019) 381(21):2020-31.

2. Paz-Ares L, Ciuleanu TE, Cobo M, Schenker M, Zurawski B, Menezes J, et al. First-line nivolumab plus ipilimumab combined with two cycles of chemotherapy in patients with non-small-cell lung cancer (CheckMate 9LA): an international, randomised, open-label, phase 3 trial. *Lancet Oncol* (2021) 22(2):198-211.

| **Supplementary Table 4** Sensitivity analyses base on the CheckMate 9LA trial | | | | | |
| --- | --- | --- | --- | --- | --- |
|  | Total cost | Life years | QALYs | ICER  ($/life year) | ICER  ($/QALY) |
| All patients | | | | | |
| Chemotherapy | $123,573 | 1.20 | 0.91 | -- | -- |
| N+I | $257,627 | 1.52 | 1.22 | weakly dominated | weakly dominated |
| N+I+chemotherapy | $357,041 | 1.92 | 1.54 | 321,246 | 365,303 |
| Patients with PD-L1 ≥ 1% | | | | | |
| Chemotherapy | $127,671 | 1.22 | 0.92 | -- | -- |
| N+I | $259,937 | 1.45 | 1.18 | weakly dominated | weakly dominated |
| N+I+chemotherapy | $342,247 | 1.73 | 1.40 | 415,938 | 450,490 |
| Patients with PD-L1 < 1% | | | | | |
| Chemotherapy | $117,183 | 1.16 | 0.87 | -- | -- |
| N+I | $252,996 | 1.72 | 1.35 | 239,906 | 282,537 |
| N+I+chemotherapy | $353,285 | 2.04 | 1.62 | 321,803 | 371,421 |
| ICER, incremental cost-effectiveness ratio; N+I, nivolumab plus ipilimumab; PD-L1, programmed death-ligand 1; QALY, quality-adjusted life year. | | | | | |
